# The Effectiveness of Serious Games in Improving Memory Among Older Adults With Cognitive Impairment: Systematic Review and Meta-analysis

**DOI:** 10.2196/35202

**Published:** 2022-08-09

**Authors:** Alaa Abd-alrazaq, Dari Alhuwail, Eiman Al-Jafar, Arfan Ahmed, Farag Shuweihdi, Shuja Mohd Reagu, Mowafa Househ

**Affiliations:** 1 AI Center for Precision Health, Weill Cornell Medicine-Qatar Doha Qatar; 2 Information Science Department, Kuwait University Kuwait Kuwait; 3 Health Informatics Unit, Dasman Diabetes Institute Kuwait Kuwait; 4 Kuwait Health Informatics Association Kuwait Kuwait; 5 Leeds Institute of Health Sciences School of Medicine University of Leeds Leeds United Kingdom; 6 Mental Health Services Hamad Medical Corporation Doha Qatar; 7 Division of Information and Computing Technology College of Science and Engineering Hamad Bin Khalifa University, Qatar Foundation Doha Qatar

**Keywords:** serious games, cognitive training, exergames, mild cognitive impairment, Alzheimer disease, dementia, memory, systematic reviews, meta-analysis, mobile phone

## Abstract

**Background:**

Memory, one of the main cognitive functions, is known to decline with age. Serious games have been used for improving memory in older adults. The effectiveness of serious games in improving memory has been assessed by many studies. To draw definitive conclusions about the effectiveness of serious games, the findings of these studies need to be pooled and aggregated.

**Objective:**

This study aimed to assess the effectiveness of serious games in improving memory in older adults with cognitive impairment.

**Methods:**

A systematic review of randomized controlled trials was carried out. The search sources included 8 databases, the reference lists of the included studies and relevant reviews, and the studies that cited the included studies. In total, 2 reviewers (AA and MH) independently carried out the study selection, data extraction, risk of bias assessment, and quality of evidence appraisal. Extracted data were synthesized using a narrative approach and a statistical approach (ie, multilevel meta-analysis), as appropriate.

**Results:**

Of the 618 citations retrieved, 18 (2.9%) met the eligibility criteria for this review. Of these 18 studies, 15 (83%) randomized controlled trials were included in 10 multilevel meta-analyses. We found that serious games were more effective than no or passive interventions in improving nonverbal memory (*P*=.02; standardized mean difference [SMD]=0.46, 95% CI 0.09-0.83) and working memory (*P*=.04; SMD=0.31, 95% CI 0.01-0.60) but not verbal memory (*P*=.13; SMD=0.39, 95% CI −0.11 to 0.89). The review also showed that serious games were more effective than conventional exercises in improving verbal memory (*P*=.003; SMD=0.46, 95% CI 0.16-0.77) but not nonverbal memory (*P*=.30; SMD=−0.19, 95% CI −0.54 to 0.17) or working memory (*P*=.99; SMD=0.00, 95% CI −0.45 to 0.45). Serious games were as effective as conventional cognitive activities in improving verbal memory (*P*=.14; SMD=0.66, 95% CI −0.21 to 1.54), nonverbal memory (*P*=.94; SMD=−0.01, 95% CI −0.32 to 0.30), and working memory (*P*=.08; SMD=0.37, 95% CI −0.05 to 0.78) among older adults with cognitive impairment. Finally, the effect of adaptive serious games on working memory was comparable with that of nonadaptive serious games (*P*=.08; SMD=0.18, 95% CI −0.02 to 0.37).

**Conclusions:**

Serious games have the potential to improve verbal, nonverbal, and working memory in older adults with cognitive impairment. However, our findings should be interpreted cautiously given that most meta-analyses were based on a few studies (≤3) and judged to have a low quality of evidence. Therefore, serious games should be offered as a supplement to existing proven and safe interventions rather than as a complete substitute until further, more robust evidence is available. Future studies should investigate the short- and long-term effects of serious games on memory and other cognitive abilities among people of different age groups with or without cognitive impairment.

## Introduction

### Background

Life expectancy has increased worldwide as people have better access to health care services and an improved standard of living. As a result, people are living longer [1-3]. According to the United Nations World Population Aging 2020 report [4], the number of people aged ≥65 years has increased up to 727 million worldwide. The older population group is expected to increase to 16% by 2050 compared with 9.3% in 2020 [4]. The older population group is more likely to develop cognitive impairment [5,6], which is a decline in cognitive abilities and functions such as memory, attention, concentration, learning, and language [7,8]. According to the Alzheimer’s Association, approximately 12% to 18% of people aged ≥60 years have mild cognitive impairment (MCI) [9].

MCI refers to a decline in the ability to learn new information or recall stored information and occurs along a continuum that ranges from normal to severely impaired cognition [10]. Although inconsistencies exist in screening for MCIs, it is certain that they occur because of brain changes owing to multiple factors, including older age, injuries to the brain, diabetes, hypertension, stroke, depression, and physical inactivity [11]. Memory is one of the main cognitive functions that decline with age. Memory is known as the ability of the brain to hold information and recall it as needed. There are different types of memory: verbal, nonverbal, and working memory. Verbal memory refers to a person’s ability to remember what they read or hear of information that was already learned [12]. On the other hand, nonverbal memory refers to storing, retrieving, and remembering nonverbal information, content, or experiences, such as images, feelings, tastes, sounds, shapes, and smells [13]. Furthermore, memory is divided into 3 types according to the period for which the memorized information is retained: short-term, long-term, and working memory. Short-term memory temporarily holds a limited amount of information [14], whereas long-term memory refers to the relatively permanent storage and recall of information [15]. Working memory refers to the temporary storage of a limited amount of information to be used in the execution of cognitive activities such as learning, reasoning, and comprehension [16].

Several nonpharmacological interventions can be used to improve memory, such as physical exercise, cognitive behavioral therapy, psychosocial therapy, good nutrition, and serious games [17]. Serious games are defined as electronic games that are played for purposes beyond leisure to promote the users’ mental, physical, and social well-being [18,19]. Recent evidence suggests that exergames are effective in improving physical and cognitive function in people with MCIs [20] as well as their compliance and adherence to medical interventions embedded in serious games [21,22]. Previous systematic reviews have shown that serious games have the potential to prevent or alleviate mental disorders such as depression [23], anxiety [24], and cognitive impairment [25]. Several types of serious games have been used to improve cognitive abilities, namely (1) cognitive training games (which deliver cognitive activities to maintain or improve cognitive functions) and (2) exergames (which entail physical exercises as part of the intended gameplay [25]). Compared with conventional exercise and cognitive training, serious games can positively affect mood, social functioning, mental health well-being, and cognitive flexibility in older adults [26-29].

### Research Problem and Objectives

The effectiveness of serious games in improving memory has been assessed by many studies. To draw definitive conclusions about the effectiveness of serious games, the findings of these studies need to be pooled and aggregated. Several systematic reviews have summarized the evidence from these studies; however, they had a different aim and scope from this review. Specifically, these reviews (1) focused on healthy older adults and not necessarily those with cognitive impairment [17,30-33] (therefore, future reviews should consider older adults with cognitive impairment), (2) included pilot randomized controlled trials (RCTs) and quasi-experiments [17,20,33,34] (thus, future reviews should include only RCTs), (3) performed an outdated search (>5 years [17,32,34]; therefore, an updated review or a new review are required), (4) did not assess the quality of evidence [17,20,30,33,34] (thus, the quality of the evidence should be assessed in future reviews), (5) only focused on a specific type of serious game such as cognitive training games [30,34] and exergames [17,20,33] (hence, future reviews should consider all types of serious games), (6) focused on a certain type of memory (working memory [34]; therefore, all types of memory should be considered in upcoming reviews), or (7) did not compare the effect of serious games with a specific comparator (eg, no intervention, conventional exercises, or conventional cognitive activities [17,20,30,33,34]; thus, further reviews are needed to compare the effect of serious games with a specific comparator). To address the aforementioned gaps, this study aimed to assess the effectiveness of serious games in improving memory among older adults with cognitive impairment. This review focused only on memory as other cognitive domains—for example, global cognition [25], executive functions [35], and processing speed [36]—were targeted by previous reviews.

## Methods

The authors followed the expanded version of the PRISMA (Preferred Reporting Items for Systematic Reviews and Meta-Analyses) guidelines to conduct a systematic review and meta-analyses (Multimedia Appendix 1). The protocol for this review was registered with PROSPERO (CRD42021292150).

### Eligibility Criteria

This review included only RCTs that looked at the effectiveness of serious games in improving memory in older adults with cognitive impairment. The target intervention in this review was serious games supplied on any digital platform, such as computers (PCs), consoles (Xbox and PlayStation), mobile phones, handheld devices, Nintendo, or any other computerized device. Furthermore, components of gaming had to be used as an important and major technique for reaching the intervention’s goal. Serious games had to be used solely for the purpose of therapy. Studies combining serious games with other interventions were eligible if the control group received the same adjacent intervention. Nondigital games and those used for other purposes, such as monitoring, screening, diagnosis, and research, were excluded.

The study focused on the older adult population (aged ≥60 years) who had any type of cognitive impairment or condition (eg, MCI, Alzheimer disease, or dementia). Their diagnosis had to be confirmed by checking the inclusion criteria or baseline scores against standardized diagnostic criteria (eg, Mini-Mental State Examination and Montreal Cognitive Assessment). This review did not focus on healthy older adults, health care providers, or caregivers. No restrictions were applied regarding sex and ethnicity.

The main outcome of interest in this review was memory regardless of the type (verbal, nonverbal, or working memory) and regardless of the tool used for measuring the outcome. Studies were excluded if they assessed only other cognitive outcomes (eg, language and processing speed), cost-effectiveness, acceptance, feasibility, or satisfaction. This review focused on outcome data that were measured immediately after the intervention rather than on follow-up data.

Only RCTs conducted in English and from 2010 onward were considered. Pilot or feasibility RCTs, quasi-experiments, observational studies, and reviews were omitted. Studies published as journal articles, conference proceedings, or dissertations were included. Reviews, conference abstracts, proposals, editorials, and commentaries were all excluded. Finally, no restrictions related to the country of publication, comparator, or study setting were applied.

### Information Sources and Search Strategy

The studies that were relevant to this review were found by searching 7 bibliographic databases: MEDLINE (via Ovid), PsycINFO (via Ovid), EMBASE (via Ovid), CINAHL (via EBSCO), IEEE Xplore, ACM Digital Library, and Scopus. Furthermore, we searched the search engine Google Scholar. Owing to the high number of papers obtained through Google Scholar, only the first 10 pages (ie, 100 records) were taken into account as they were automatically ordered based on their relevance [37]. The first author (AA) conducted the search on August 6, 2021. An automatic alert was set up to retrieve studies that were added to the databases after that date; this continued for 16 weeks (ending on December 5, 2021). Forward reference list checking (ie, screening studies that cited the included studies) and backward reference list checking (ie, screening the reference lists of the included studies and relevant reviews) were carried out to retrieve further studies.

To develop the search query for this review, the authors consulted 2 experts in digital mental health and checked the search queries used in other systematic reviews within this field. The chosen search terms were related to the target population (eg, cognitive impairment), target intervention (eg, serious games and exergames), and target study design (eg, RCTs). Multimedia Appendix 2 summarizes the search query that was used for searching each of the 8 databases.

### Selection Process

Relevant studies were identified taking the following steps. First, the obtained studies were imported into EndNote X8 (Clarivate Analytics) to identify and delete duplicate items. Second, the titles and abstracts of all the retrieved studies were evaluated in the second phase by 2 reviewers (AA and MH) working independently. Finally, the 2 reviewers independently evaluated the entire texts of the studies included in the previous step. Any disagreements in the 2 previous steps were resolved via discussion. The interrater agreement (Cohen κ) in steps 2 and 3 was 0.94 and 0.96, respectively, indicating a near-perfect level of interrater agreement [38].

### Data Collection Process

In total, 2 independent reviewers (AA and MH) used Microsoft Excel to extract data from all the included studies. The data extraction form used to extract data from the included studies was pilot-tested using 2 of the included studies (Multimedia Appendix 3). The reviewers’ disagreements were resolved through discussion. An interrater agreement of 0.85 was observed, indicating a near-perfect degree of agreement. If data such as the mean, SD, and sample size were unavailable from the published studies, contact was made with the first and corresponding authors in an attempt to retrieve them.

### Study Risk of Bias Assessment

The Cochrane Collaboration recommends assessing the risk of bias via 2 independent reviewers (AA and MH) using the Risk of Bias 2 (RoB 2) tool [39]; as such, these guidelines were followed for this review. The RoB 2 tool assesses the risk of bias in 5 domains of RCTs: randomization process, deviations from intended interventions, missing outcome data, measurement of the outcome, and selection of the reported result [39]. The risk of bias judgments in these domains were used to determine the overall risk of bias of each included study. Any inconsistencies in decisions between the reviewers were resolved by consulting a third reviewer. Interrater agreement between the reviewers was near perfect (Cohen κ=0.93) [38].

### Synthesis Methods

A narrative and statistical approach was used to synthesize the information acquired. In our narrative synthesis, we used texts and tables to describe the characteristics of the included studies (demographic, intervention, comparator, and outcome variables). The results of the experiments were categorized and pooled based on measured outcome (ie, verbal, nonverbal, and working memory) and the comparator (ie, control, conventional exercises, conventional cognitive training, and other serious games). A meta-analysis was conducted when at least two studies with the same measured outcome and comparator submitted enough data (ie, mean, SD, and number of participants in each intervention group). Owing to the type of data for the outcome of interest (memory) being continuous and the methods used to measure the outcome being variable throughout the included studies, the standardized mean difference (SMD; Cohen *d*) was used to analyze the overall effect of each study. The random effects model was used for the analysis because of the high clinical heterogeneity among the meta-analyzed studies in terms of serious game characteristics (eg, type, duration, frequency, and period), population characteristics (eg, sample size, mean age, and health condition), and outcome measures (ie, tools and follow-up period). As several studies used more than one outcome measure to assess memory, the dependency on effect sizes within or across studies will be introduced in the meta-analysis. As a result, a multilevel meta-analysis considering the dependency on effect sizes and sampling covariance between the effect sizes was used [40-42]. Namely, the multilevel meta-analysis should be applied when effect sizes within the same study are very likely to be more similar to each other than the effect sizes across studies [42]. The *R* (version 4.3.1; R Foundation for Statistical Computing) statistical package was used to perform the analysis. We used the function *rma.mv* in the library *metafor*, which is a library in *R*, to perform the multilevel meta-analysis [43].

If we observed a statistically significant difference between the groups in a meta-analysis, we further sought to examine if it was clinically significant. The phrase “minimal clinically important difference” (MCID) refers to the smallest change in a measured outcome that a patient would consider worthwhile and significant enough to warrant a change in treatment. The MCID boundaries were calculated as 0.5 times the SMD of the meta-analyzed studies.

We calculated *I*^2^ and a chi-square *P* value to investigate the degree and statistical significance of the heterogeneity in the meta-analyzed studies, respectively. A chi-square *P* value of ≤.05 suggests heterogeneous meta-analyzed studies [44]. When *I*^2^ ranged from 0% to 40%, 30% to 60%, 50% to 90%, and 75% to 100%, the degree of heterogeneity was judged to be insignificant, moderate, substantial, or considerable, respectively [44].

### Certainty of Evidence

To appraise the overall quality of evidence resulting from the meta-analyses, we applied the Grading of Recommendations Assessment, Development, and Evaluation approach [45], which assesses the quality of evidence based on 5 domains: risk of bias, inconsistency (ie, heterogeneity), indirectness, imprecision, and publication bias [45]. In total, 2 reviewers independently rated the overall quality of the meta-analyzed evidence, and disagreements were resolved through discussion. The interrater agreement of the reviewers was considered near perfect (Cohen κ=0.87) [38].

## Results

### Study Selection

**Figure 1:**
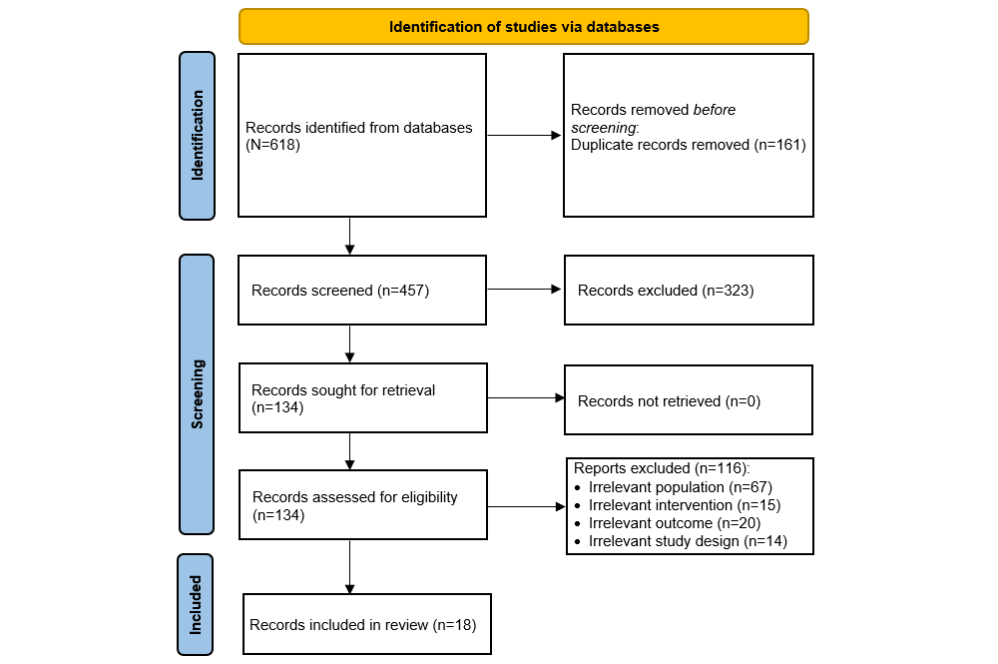
Flowchart of the study selection process.

The total number of records retrieved by searching the predefined databases was 618 (Figure 1). Of these 618 records, 161 (26.1%) duplicates were removed using the EndNote software. Checking titles and abstracts of the remaining records led to the exclusion of 52.3% (323/618). After reading the full texts of the remaining 134 publications, 116 (86.6%) were excluded, mainly because of the population (n=67, 57.8%). The list of studies that were excluded after screening the full texts is provided in Multimedia Appendix 4. No additional studies were found through backward and forward reference list checking. In total, 18 RCTs were included in this review [46-63]. Of these 18 studies, 15 (83%) were included in 10 meta-analyses [47-49,51,52,54-63]. A total of 17% (3/18) of the studies were excluded from the meta-analyses because 33% (1/3) [46] did not report the data required for the meta-analysis (eg, mean and SD) and 67% (2/3) [53,61] compared serious games with other serious games that had different characteristics; therefore, including them in a meta-analysis would not make sense.

### Study Characteristics

The included studies were published between 2012 and 2021 (Table 1). The year in which the largest number of included studies was published was 2015 (4/18, 22%). The included studies were carried out in 13 different countries, and there was a general equal distribution of studies in these countries. All the included studies were peer-reviewed journal articles except for a book chapter included (1/18, 6%). The trial type was parallel RCT in most of the included studies (17/18, 94%).

The sample size of the included studies varied from 20 to 209, with an average of 81. The mean age of the participants in the included studies ranged from 66 to 83.1 years, with an average of 74.5 years. The percentage of men in the included studies ranged from 21.5% to 71%, with an average of 46.5%. The participants in most of the included studies had MCI (14/18, 78%). Participants were recruited from clinical settings in 67% (12/18) of the studies, from the community in 28% (5/18) of the studies, and from both clinical settings and the community in 6% (1/18) of the studies.

Serious games alone were used as interventions in 89% (16/18) of the included studies, whereas the remaining 11% (2/18) of the studies used serious games combined with conventional exercises [48] or sham exercises [49] (Table 2). The included studies used 16 different serious games. On the basis of the therapeutic modality that they delivered, the serious games used in the included studies were grouped into 2 types: cognitive training games (16/18, 89%) and exergames (2/18, 11%). Games were designed with a “serious” purpose from the beginning (designed serious games) in all studies except for 6% (1/18) that used a purpose-shifted game (which was not designed as a serious game from the start but rather was used for a serious purpose). The most common platform used for playing the games were computers (14/18, 78%). In 67% (12/18) of the studies, serious games were played under the supervision of health care providers or caregivers. The duration of the games in the included studies ranged from 7 to 90 minutes, and the most common duration was 60 minutes (7/18, 39%). The frequency of playing the games varied between 2 and 7 times per week, but it was 2 times per week in half of the studies (9/18, 50%). The period of intervention ranged from 2 to 25 weeks, but it was ≤12 weeks in 72% (13/18) of the studies.

The comparison groups received only passive interventions in 39% (7/18) of the studies, whereas they received only active interventions in 44% (8/18) of the studies (eg, conventional exercises and conventional cognitive activities; Table 3). In total, 17% (3/18) of the studies delivered both active and passive interventions as comparators. The duration of the active comparators ranged from 7 to 100 minutes. The frequency of the active comparators varied between 2 and 7 times per week. The period of the active comparators varied between 2 and 25 weeks. Most of the included studies (16/18, 89%) measured more than one outcome. The measured outcomes were verbal memory in 78% (14/18) of the studies, nonverbal memory in 61% (11/18) of the studies, and working memory in 67% (12/18) of the studies. The studies used 32 different tools to measure these outcomes, but the most common tool used was the Wechsler Memory Scale Third Edition (7/18, 39%). The outcomes were measured immediately after the intervention in all the included studies (18/18, 100%). The follow-up period ranged from 4 to 264 weeks. Participant attrition was reported in 89% (16/18) of the studies, and it ranged from 0 to 23.

**Table 1:** Characteristics of the studies and populations (N=18).

Study	Year	Country	Publication type	RCT^a^ type	Sample size	Age, mean	Sex (male; %)	Health condition	Setting
Valdes et al [46]	2012	United States	Journal article	Parallel	195	77.7	33.3	MCI^b^	Clinical
Zhuang et al [47]	2013	China	Journal article	Parallel	33	83.1	24.2	MCI; dementia	Clinical
Hagovská et al [48]	2016	Slovakia	Journal article	Parallel	80	67	51.2	MCI	Clinical
Singh et al [49]	2014	Australia	Journal article	Factorial	100	70.1	32	MCI	Community
Gooding et al [50]	2016	United States	Journal article	Parallel	96	75.6	58.1	MCI	Clinical
Liao et al [51]	2021	Taiwan	Journal article	Parallel	61	81.5	32.6	MCI	Community
Finn and McDonald [52]	2015	Australia	Journal article	Parallel	31	75.6	71	MCI	Clinical
Park and Park [53]	2017	South Korea	Journal article	Parallel	78	67.3	53.8	MCI	Community
Cavallo et al [54]	2016	Italy	Journal article	Parallel	80	76.4	36.3	AD^c^	Clinical
Leung et al [55]	2015	Hong Kong	Journal article	Parallel	209	70.1	21.5	MCI	Community
Yang and Kwak [56]	2017	South Korea	Journal article	Parallel	20	71	70	AD	Clinical
Tarnanas et al [57]	2014	Greece	Book chapter	Parallel	114	70.3	39	MCI	Clinical
Flak et al [58]	2019	Norway	Journal article	Parallel	85	66	66.7	MCI	Clinical
Herrera et al [59]	2012	France	Journal article	Parallel	22	76.6	50	MCI	Clinical
Savulich et al [60]	2017	United Kingdom	Journal article	Parallel	42	76.1	59.5	MCI	Clinical
Boller et al [61]	2012	France	Journal article	Parallel	36	81.2	36.1	AD	Clinical
Karssemeijer et al [62]	2019	Netherlands	Journal article	Parallel	115	79.9	53.9	Dementia	Clinical, community
Hyer et al [63]	2015	United States	Journal article	Parallel	68	75.2	47.1	MCI	Community

^a^RCT: randomized controlled trial.

^b^MCI: mild cognitive impairment.

^c^AD: Alzheimer disease.

**Table 2:** Characteristics of the interventions (N=18).

Study	Serious game name	Serious game type	Platform	Supervision	Duration (minutes)	Frequency (times per week)	Period (weeks)
Valdes et al [46]	SOPT	Cognitive training game	PC	Supervised	60	2	5
Zhuang et al [47]	NR^a^	Cognitive training game	PC	Supervised	75	3	24
Hagovská et al [48]	CogniPlus	Cognitive training game	PC	Supervised and unsupervised	30	2	10
Singh et al [49]	COGPACK	Cognitive training game	PC	Supervised	75	2	25
Gooding et al [50]	BrainFitness	Cognitive training game	PC	Supervised and unsupervised	60	2	17
Liao et al [51]	Tano and LongGood	Exergame	Kinect, VR^b^ headset	Supervised	60	3	12
Finn and McDonald [52]	E-Prime	Cognitive training game	PC	Supervised	NR	2	4
Park and Park [53]	CoTras	Cognitive training game	PC	Supervised	30	3	10
Cavallo et al [54]	Brainer	Cognitive training game	PC	Supervised	30	3	12
Leung et al [55]	BrainFitness	Cognitive training game	PC	Unsupervised	60	3	13
Yang and Kwak [56]	Brain-Care	Cognitive training game	PC	Unsupervised	60	2	12
Tarnanas et al [57]	Virtual Reality Museum	Cognitive training game	VR headset	Supervised	90	2	21
Flak et al [58]	Cogmed	Cognitive training game	PC	Unsupervised	30-40	5	5
Herrera et al [59]	NR	Cognitive training game	PC	Supervised	60	2	12
Savulich et al [60]	Game Show	Cognitive training game	Tablet	Supervised	60	2	4
Boller et al [61]	NR	Cognitive training game	PC	Supervised	7-10	3	2
Karssemeijer et al [62]	NR	Exergame	Stationary bicycle and screen	Supervised	30-50	3	12
Hyer et al [63]	Cogmed	Cognitive training game	PC	Supervised and unsupervised	40	7	5-7

^a^NR: not reported.

^b^VR: virtual reality.

**Table 3:** Characteristics of the comparators and outcomes (N=18).

Study	Comparator	Duration (minutes)	Frequency (times per week)	Period (weeks)	Measured outcomes	Outcome measures	Follow-up	Attrition, N
Valdes et al [46]	Control	N/A^a^	N/A	N/A	VM^b^	HVLT^c^; RAVLT^d^; RBMT^e^	After the intervention; 52-, 104-, 156-, and 261-week follow-up	NR^f^
Zhuang et al [47]	Control	N/A	N/A	N/A	VM	ACE-R^g^	After the intervention	10
Hagovská et al [48]	Conventional exercises	30	7	10	VM	ACE-R	After the intervention	2
Singh et al [49]	Conventional exercises+sham cognitive training; serious games+conventional exercises; control	Conventional exercises+sham cognitive training: 75; serious games+conventional exercises: 100; control: 60	2	25	VM; NVM^h^	BVRT-R^i^; WMS-III-LM^j^	After the intervention; 74-week follow-up	14
Gooding et al [50]	Empirically validated serious game; commercially available serious game	60	2	17	VM; NVM	WMS-R-VR-II^k^; WMS-R-LM^l^; BSRT^m^	After the intervention	22
Liao et al [51]	Conventional exercises	60	3	12	VM; WM^n^	CVLT^o^; SBTT^p^	After the intervention	15
Finn and McDonald [52]	Control	NR	2	4	VM; WM	WMS-IV-VPA-II^q^; WMS-IV-SS^r^	After the intervention	7
Park and Park [53]	Commercially available exergame	30	3	10	VM; WM	RAVLT; WAIS-DSB^s^	After the intervention	0
Cavallo et al [54]	Control	N/A	N/A	N/A	VM; NVM; WM	RBMT; WMS-R-DSB^t^; TSWRT^u^	After the intervention; 24-week follow-up	4
Leung et al [55]	Control	60	3	13	VM; NVM; WM	WMS-III-FP^v^; WMS-III-LM; WMS-III-DST^w^; WMS-III-VSST^x^	After the intervention	0
Yang and Kwak [56]	Control	N/A	N/A	N/A	VM; NVM; WM	ROCFT^y^; SVLT^z^; WMS-III-DSB^aa^	After the intervention	0
Tarnanas et al [57]	Control; conventional cognitive activities	90	2	21	VM; NVM; WM	ROCFT; RAVLT; WMS-III-DSB	After the intervention	9
Flak et al [58]	Nonadaptive serious game	30 to 40	5	5	VM; NVM; WM	ROCFT; WMS-III-FII^ab^; WMS-III-LM; CVLT-II^ac^; WMS-III-DSB; WMS-III-SS^ad^; WMS-III-LNS^ae^	After the intervention; 4- and 16-week follow-up	17
Herrera et al [59]	Conventional cognitive activities	60	2	12	VM; NVM; WM	ROCFT-R^af^; BEM-WLTR^ag^; MMSE-R^ah^; WMS-R-DSB	After the intervention; 24-week follow-up	NR
Savulich et al [60]	Control	N/A	N/A	N/A	NVM	BVRT-R	After the intervention	0
Boller et al [61]	Serious game; control	7 to 10	3	2	NVM; WM	SRT^ai^; n-BT^aj^; RST^ak^	After the intervention	0
Karssemeijer et al [62]	Conventional exercises (aerobic exercises); conventional exercises (relaxation and flexibility exercises)	30 to 50	3	12	NVM; WM	LLT-R^al^; WAIS-III-DS^am^; WMS-III-VSST	After the intervention; 24-week follow-up	23
Hyer et al [63]	Nonadaptive serious game	40	7	5 to 7	WM	WMS-III-DST; WMS-III-LNS	After the intervention; 12-week follow-up	9

^a^N/A: not applicable.

^b^VM: verbal memory.

^c^HVLT: Hopkins Verbal Learning Test.

^d^RAVLT: Rey Auditory Verbal Learning Test.

^e^RBMT: Rivermead Behavioral Memory Test.

^f^NR: not reported.

^g^ACE-R: Addenbrooke Cognitive Examination-Revised.

^h^NVM: nonverbal memory.

^i^BVRT-R: Benton Visual Retention Test-Revised, Fifth Edition.

^j^WMS-III-LM: Wechsler Memory Scale Third Edition-Logical Memory.

^k^WMS-R-VR-II: Wechsler Memory Scale-Revised-Visual Reproductions II.

^l^WMS-R-LM: Wechsler Memory Scale-Revised-Logical Memory.

^m^BSRT: Buschke Selective Reminding Test.

^n^WM: working memory.

^o^CVLT: California Verbal Learning Test.

^p^SBTT: spatial n-back task test.

^q^WMS-IV-VPA-II: Wechsler Memory Scale Fourth Edition-Verbal Paired Associates II.

^r^WMS-IV-SS: Wechsler Memory Scale Fourth Edition-Symbol Span.

^s^WAIS-DSB: Wechsler Adult Intelligence Scale-Digit Span Backwards.

^t^WMS-R-DSB: Wechsler Memory Scale-Revised-Digit Span Backwards.

^u^TSWRT: two-syllable word repetition test.

^v^WMS-III-FP: Wechsler Memory Scale Third Edition-Family Pictures.

^w^WMS-III-DST: Wechsler Memory Scale Third Edition-Digit Span Test.

^x^WMS-III-VSST: Wechsler Memory Scale Third Edition-Visual-Spatial Span Test.

^y^ROCFT: Rey-Osterrieth complex figure test.

^z^SVLT: Seoul Verbal Learning Test.

^aa^WMS-III-DSB: Wechsler Memory Scale Third Edition-Digit Span Backwards Test.

^ab^WMS-III-FII: Wechsler Memory Scale Third Edition-Faces II.

^ac^CVLT-II: California Verbal Learning Test-Second Edition.

^ad^WMS-III-SS: Wechsler Memory Scale Third Edition-Symbol Span.

^ae^WMS-III-LNS: Wechsler Memory Scale Third Edition-Letter-Number Sequencing.

^af^ROCFT-R: Rey-Osterrieth complex figure test-Revised.

^ag^BEM-WLTR: Batterie d’Efficience Mnesique-word list total recall.

^ah^MMSE-R: Mini-Mental State Examination-Recall.

^ai^SRT: source recognition task.

^aj^n-BT: n-back task.

^ak^RST: reading span task.

^al^LLT-R: Location Learning Test-Revised.

^am^WAIS-III-DS: Wechsler Adult Intelligence Scale Second Edition-Digit Span.

### Risk of Bias in the Studies

**Figure 2:**
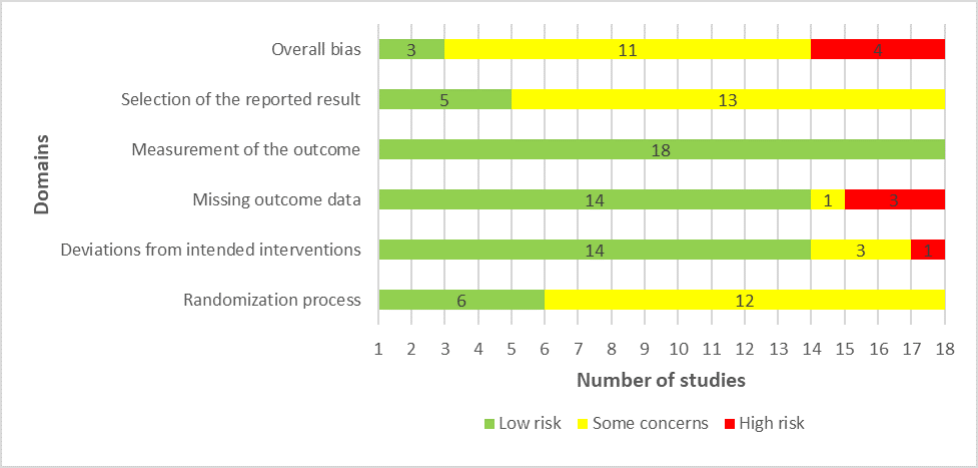
Review authors’ judgments about each “risk of bias” domain.

An appropriate random allocation sequence for the randomization process was used in 44% (8/18) of the studies. Researchers in 39% (7/18) of the studies concealed the allocation sequence until participants were assigned to the interventions. The groups were comparable at baseline in all studies (18/18, 100%). Thus, the risk of bias owing to the randomization process was rated as low in only 33% (6/18) of the studies (Figure 2).

Participants and those who delivered the interventions were aware of the assigned interventions during the trial in 67% (12/18) and 83% (15/18) of the studies, respectively. None of the studies reported a deviation from the intended intervention because of experimental contexts; however, 11% (2/18) of the studies provided insufficient information to verify if protocol deviations had occurred. Appropriate analysis methods (eg, intention-to-treat analysis) were used in 89% (16/18) of the studies to estimate the effect of the intervention. According to these judgments, the risk of bias because of deviations from the intended interventions was low in 78% (14/18) of the studies (Figure 2).

Missing outcome data were <5% in 44% (8/18) of the studies. There was evidence that the findings were not biased by missing outcome data in only 6% (1/18) of the studies. The missing outcome data resulted from reasons that were documented and not related to the outcome in 28% (5/18) of the studies. Therefore, there was a low risk of bias because of missing outcome data in 78% (14/18) of the studies (Figure 2).

In all the included studies (18/18, 100%), the outcomes of interest were evaluated using appropriate measures, and the measurement methods were comparable across the intervention groups. The assessor of the outcome was aware of the assigned interventions in 39% (7/18) of the studies, but it was unlikely that the assessment of the outcome was influenced by knowledge of the intervention received in these studies. Accordingly, all studies (18/18, 100%) had a low risk of bias in the “measuring the outcome” domain (Figure 2).

In total, 28% (5/18) of the studies published their protocols in sufficient detail. In all studies (18/18, 100%), the reported outcome measurements did not differ from those specified in the analysis plan, and there was no evidence that the studies selected their results from many results produced from multiple eligible analyses of the data. On the basis of these judgments, the risk of bias because of the selection of the reported results was considered low in 28% (5/18) of the studies (Figure 2).

In the last domain, “overall bias,” the risk of bias was considered high in 22% (4/18) of the studies as they were judged as having a high risk of bias in at least one domain. A total of 61% (11/18) of the studies raised some concerns in the domain of overall bias as they had some issues in at least one of the domains and were not at high risk for any domain. The remaining 17% (3/18) of the studies were judged to be at low risk of bias for the domain of overall bias given that they were rated to be at low risk of bias for all domains. The reviewers’ judgments about each “risk of bias” domain for each included study are presented in Multimedia Appendix 5 [46-63].

### Results of the Studies

#### Overview

As mentioned earlier, the included studies assessed the effect of serious games on 3 outcomes: verbal, nonverbal, and working memory. The results of the included studies were divided into 3 groups based on these outcomes. Furthermore, the results for each outcome were grouped based on the comparator used in the studies (ie, control [no or passive interventions], conventional exercises, conventional cognitive activities, and other serious games).

#### Verbal Memory

##### Serious Games Versus Control

**Figure 3:**
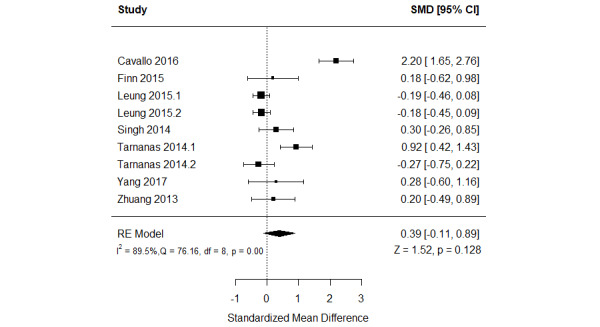
Forest plot of 7 studies (9 comparisons) comparing the effect of serious games with that of control on verbal memory. RE: random effect; SMD: standardized mean difference [49,51,54,56-59].

The effect of serious games on verbal memory was compared with that of no or passive interventions in 44% (8/18) of the studies [46,47,49,52,54-57]. A total of 13% (1/8) of these studies were not included in the meta-analysis given that they did not report the required data and we could not obtain them when contacting the authors. Of the 7 studies included in the meta-analysis, 2 (29%) assessed verbal memory using 2 different measures [55,57]. Therefore, we included the results of all these measures in the meta-analysis to form 9 comparisons (Figure 3 [47,49,52,54-57]). The meta-analysis showed no statistically significant difference (*P*=.13) in verbal memory between the serious game and control groups (SMD=0.39, 95% CI −0.11 to 0.89). The statistical heterogeneity of the evidence was considerable (*P*<.001; *I*^2^=89.5%). The high heterogeneity may be attributed to differences in sample size, participants’ health condition, period of the intervention, and outcome measures among the studies included in this analysis. The quality of the evidence was very low as it was downgraded by 5 levels owing to a high risk of bias, heterogeneity, and imprecision (Multimedia Appendix 6).

We conducted subgroup analyses, also known as moderator analyses [64], to investigate whether different characteristics of the population (ie, sample size, health condition, and recruitment setting) and intervention (ie, delivery method, duration, frequency, and period) moderated the effect of serious games on verbal memory. As shown in Multimedia Appendix 7, there was no statistically significant difference among all characteristics of the population and intervention except for the health condition of the participants (*P*=.003) and the period of the intervention (*P*=.05).

##### Serious Games Versus Conventional Exercises

**Figure 4:**
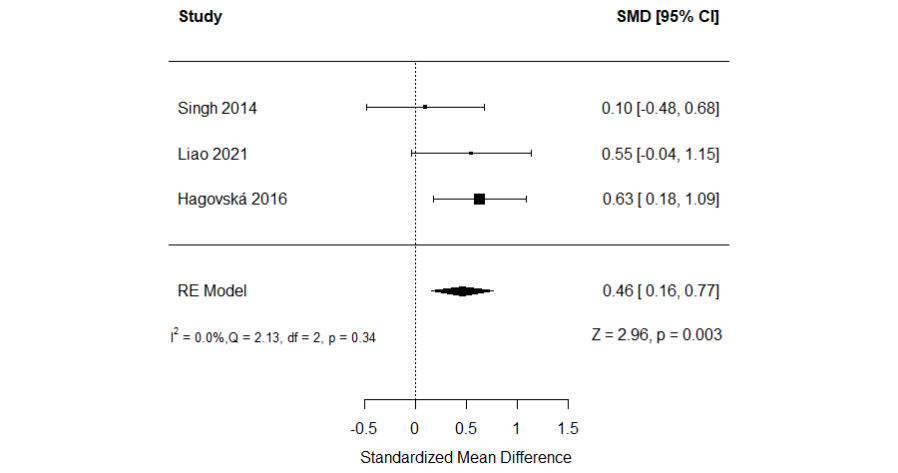
Forest plot of 3 studies comparing the effect of serious games with that of conventional exercises on verbal memory. RE: random effect; SMD: standardized mean difference [50,51,53].

The effect of serious games was compared with that of conventional exercises in 17% (3/18) of the studies [48,49,51] (Figure 4 [48,49,51]). A meta-analysis of the results of these studies showed a statistically significant difference in verbal memory (*P*=.003) between the groups, favoring serious games over conventional exercises (SMD=0.46, 95% CI 0.16-0.77). This difference was also clinically important as the overall effect was outside MCID boundaries (−0.23 to 0.23) and its 95% CI did not cross the “no effect” line (zero effect). For this outcome, the MCID boundaries were calculated as –0.5 times to +0.5 times the SMD value (0.46). The statistical heterogeneity of the evidence was not a concern (*P*=.34; *I*^2^=0%). The quality of the evidence was very low as it was downgraded by 3 levels owing to a high risk of bias and imprecision (Multimedia Appendix 6).

##### Serious Games Versus Conventional Cognitive Activities

**Figure 5:**
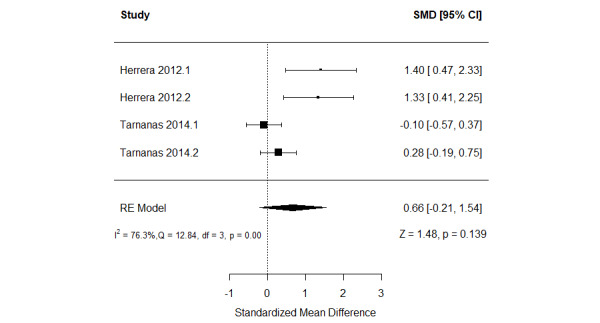
Forest plot of 2 studies (4 comparisons) comparing the effect of serious games with that of conventional cognitive activities on verbal memory. RE: random effect; SMD: standardized mean difference [59,61].

In total, 11% (2/18) of the studies examined the effect of serious games in comparison with conventional cognitive activities [57,59]. These studies assessed verbal memory using 2 different measures. Thus, we included the results of all these measures in a meta-analysis to form 4 comparisons (Figure 5 [57,59]). The meta-analysis showed no statistically significant difference (*P*=.14) in verbal memory between the groups (SMD=0.66, 95% CI −0.21 to 1.54). The statistical heterogeneity of the evidence was substantial (*P*<.001; *I*^2^=76.3%). The high heterogeneity may be attributed to differences in the platform of the intervention, period of the intervention, and outcome measures among the studies included in this analysis. The quality of the evidence was very low as it was downgraded by 5 levels owing to a high risk of bias, heterogeneity, and imprecision (Multimedia Appendix 6).

##### Serious Games Versus Other Serious Games

In total, 17% (3/18) of the studies compared the effect of serious games on verbal memory with that of other serious games [50,53,58]. Specifically, Gooding et al [50] compared the effect of a cognitive training game that included empirically validated motivational teaching and rehabilitation techniques (BrainFitnessPlus) with 2 other games: the same previous game without the aforementioned techniques (BrainFitness) and commercially available computer games and puzzles (ie, Brain Age, Sudoku, and crossword puzzles). The study found a statistically significant difference in memory between the groups, favoring BrainFitnessPlus and BrainFitness over commercially available computer games as measured by the Buschke Selective Reminding Test-Delay (BSRT-Delay) and the Wechsler Memory Scale Third Edition-Logical Memory II (WMS-III-LM-II) and favoring BrainFitness over commercially available computer games as measured by the BSRT-Delay only. However, there was no significant difference in memory between the BrainFitnessPlus and BrainFitness groups as measured by the BSRT-Delay and the WMS-III-LM-II [50].

The second trial compared the effect of a cognitive training game with that of exergames [53]. The study found no statistically significant difference (*P*=.76) in memory between the groups. The last study in this group compared the effect of a cognitive training game that adjusts the level of difficulty of the tasks based on an individual’s mastery on each level (ie, adaptive game) with the same game but without adjustment of the level of difficulty of the tasks (ie, nonadaptive game) [58]. The study showed no statistically significant difference between the groups as measured by the WMS-III-LM-II (*P*=.76) and the California Verbal Learning Test Total Hits (*P*=.30), but there was a statistically significant difference between the groups as measured by the California Verbal Learning Test II Long Delay Free Recall (*P*=.03), favoring the adaptive game over the nonadaptive game [58].

#### Nonverbal Memory

##### Serious Games Versus Control

**Figure 6:**
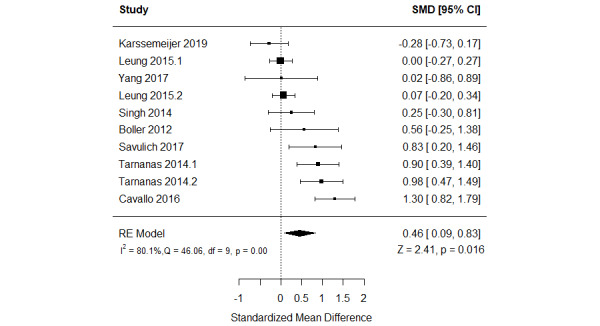
Forest plot of 8 studies (10 comparisons) comparing the effect of serious games with that of control on nonverbal memory. RE: random effect; SMD: standardized mean difference [51,56-59,62-64].

The effect of serious games on nonverbal memory was compared with that of no or passive interventions in 44% (8/18) of the studies [49,54-57,60-62]. Of these 8 studies, 2 (25%) assessed nonverbal memory using 2 different measures [55,57]. Therefore, we included the results of all these measures in the meta-analysis to form 9 comparisons (Figure 6) [49,54-57,60-62]. The meta-analysis showed a statistically significant difference (*P*=.02) in nonverbal memory between the groups, favoring serious games over no or passive interventions (SMD=0.46, 95% CI 0.09-0.83). This difference was also clinically important as the overall effect was outside MCID boundaries (−0.23 to 0.23) and its CI did not cross the “no effect” line (zero effect). For this outcome, the MCID boundaries were calculated as –0.5 times to +0.5 times the SMD value (0.46). The statistical heterogeneity of the evidence was substantial (*P*<.001; *I*^2^=80.1%). The high heterogeneity may be attributed to differences in sample sizes, participants’ health conditions, duration of the intervention, period of the intervention, platform of the intervention, and outcome measures among the studies included in this analysis. The quality of the evidence was very low as it was downgraded by 5 levels owing to a high risk of bias, heterogeneity, and imprecision (Multimedia Appendix 8). Subgroup analyses showed no statistically significant difference for all characteristics of the population and intervention (*P*>.05; Multimedia Appendix 9).

##### Serious Games Versus Conventional Exercises

**Figure 7:**
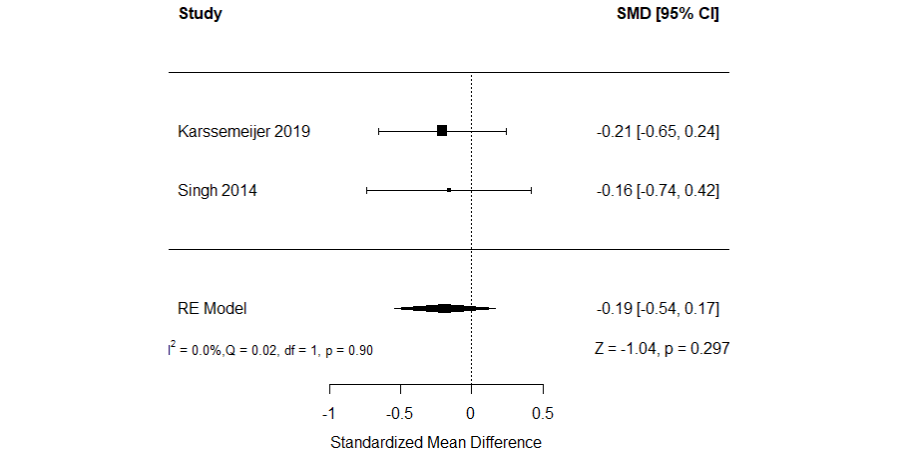
Forest plot of 2 studies comparing the effect of serious games with that of conventional exercises on nonverbal memory. RE: random effect; SMD: standardized mean difference [51,64].

The effect of serious games on nonverbal memory was compared with that of conventional exercises in 11% (2/18) of the studies [49,62]. As shown in Figure 7 [49,62], there was no statistically significant difference (*P*=.30) in nonverbal memory between the groups (SMD=−0.19, 95% CI −0.54 to 0.17). The statistical heterogeneity of the evidence was not a concern (*P*=.90; *I*^2^=0%). The quality of the evidence was very low as it was downgraded by 3 levels owing to a high risk of bias and imprecision (Multimedia Appendix 8).

##### Serious Games Versus Conventional Cognitive Activities

**Figure 8:**
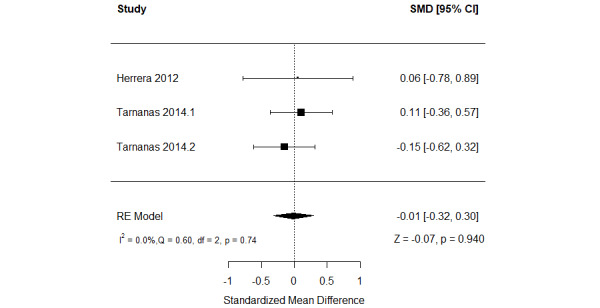
Forest plot of 2 studies (3 comparisons) comparing the effect of serious games with that of conventional cognitive activities on nonverbal memory. RE: random effect; SMD: standardized mean difference [59,61].

The effect of serious games on nonverbal memory was compared with that of conventional cognitive activities in 11% (2/18) of the studies [57,59]. Of these 2 studies, 1 (50%) assessed nonverbal memory using 2 different measures [57]. Therefore, we included the results of all these measures in the meta-analysis to form 3 comparisons (Figure 8 [57,59]). The meta-analysis showed no statistically significant difference (*P*=.94) in nonverbal memory between the groups (SMD=−0.01, 95% CI −0.32 to 0.30). The statistical heterogeneity of the evidence was not a concern (*P*=.74; *I*^2^=0%). The quality of the evidence was very low as it was downgraded by 4 levels owing to a high risk of bias and imprecision (Multimedia Appendix 8).

##### Serious Games Versus Other Serious Games

In total, 17% (3/18) of the studies compared the effect of serious games on nonverbal memory with that of other serious games [50,58,61]. Specifically, Gooding et al [50] compared the effect of BrainFitnessPlus with that of BrainFitness and commercially available computer games. The study showed no statistically significant difference in memory between any 2 of these groups [50].

The second study compared the effect of an adaptive serious game with that of a nonadaptive serious game [58]. The study showed no statistically significant difference in nonverbal memory between the groups as measured by the Rey-Osterrieth complex figure test-delayed recall (*P*=.25) and the Wechsler Memory Scale Third Edition-Faces II (*P*=.61) [58].

The last study in this group assessed the effect of 2 cognitive training games [61]. Both games consisted of a study and a test phase. In each session of the study phase, both games asked participants to read and remember 16 words presented one at a time on a computer screen for 3 seconds followed by a 1-second white screen [61]. In the test phase, participants were asked to recognize the 16 study words, which were mixed with 16 new words in the first game (recollection training game) and 32 new words in the second game (recognition practice game) [61]. The study showed no statistically significant difference (*P*=.17) in nonverbal memory between the 2 groups [61].

#### Working Memory

##### Serious Games Versus Control

**Figure 9:**
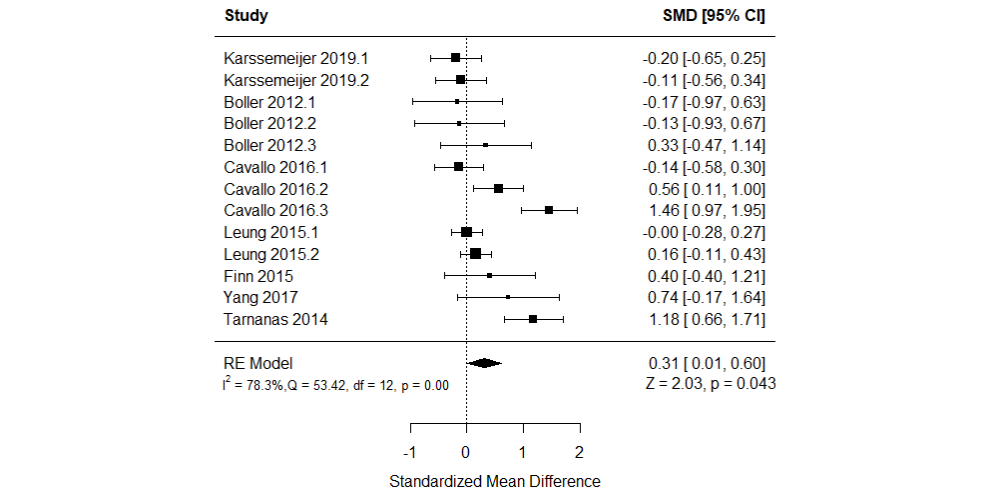
Forest plot of 7 studies (13 comparisons) comparing the effect of serious games with that of control on working memory. RE: random effect; SMD: standardized mean difference [54,56-59,63,64].

The effect of serious games on working memory was compared with that of control (no or passive interventions) in 39% (7/18) of the studies [52,54-57,61,62]. Of these 7 studies, 4 (57%) assessed working memory using more than one measure [54,55,61,62]. Therefore, we included the results of all these measures in the meta-analysis to form 13 comparisons (Figure 9) [52,54-57,61,62]. The meta-analysis showed a statistically significant difference (*P*=.04) in working memory between the groups, favoring serious games over no or passive interventions (SMD=0.31, 95% CI 0.01-0.60). This difference was also clinically important as the overall effect was outside MCID boundaries (−0.16 to 0.16) and its CI did not cross the “no effect” line (zero effect). For this outcome, the MCID boundaries were calculated as –0.5 times to +0.5 times the SMD value (0.31). The statistical heterogeneity of the evidence was substantial (*P*<.001; *I*^2^=78.3%). The high heterogeneity may be attributed to differences in sample sizes, percentage of men, participants’ health conditions, duration of the intervention, period of the intervention, and outcome measures among the studies included in this analysis. The quality of the evidence was very low as it was downgraded by 5 levels owing to a high risk of bias, heterogeneity, and imprecision (Multimedia Appendix 10). Subgroup analyses showed no statistically significant difference for all characteristics of the population and intervention (*P*>.05; Multimedia Appendix 11).

##### Serious Games Versus Conventional Exercises

**Figure 10:**
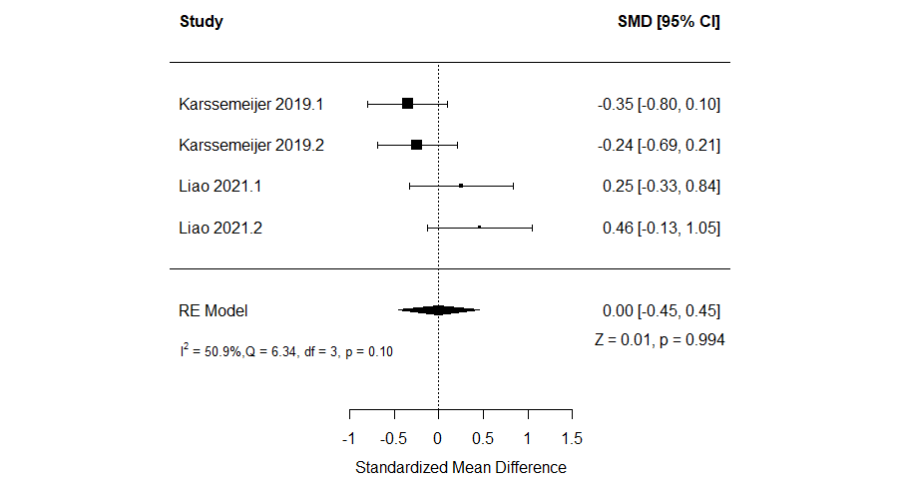
Forest plot of 2 studies (4 comparisons) comparing the effect of serious games with that of conventional exercises on working memory. RE: random effect; SMD: standardized mean difference [53,64].

The effect of serious games on working memory was compared with that of conventional exercise in 11% (2/18) of the studies [51,62]. Both studies assessed working memory using 2 different measures. Thus, we included the results of all these measures. As shown in Figure 10 [51,62], there was no statistically significant difference (*P*=.99) in working memory between the serious game and conventional exercise groups (SMD=0.00, 95% CI −0.45 to 0.45). The statistical heterogeneity of the evidence was moderate (*P*=.10; *I*^2^=50.9%). The quality of the evidence was very low as it was downgraded by 5 levels owing to a high risk of bias and imprecision (Multimedia Appendix 10).

##### Serious Games Versus Conventional Cognitive Activities

**Figure 11:**
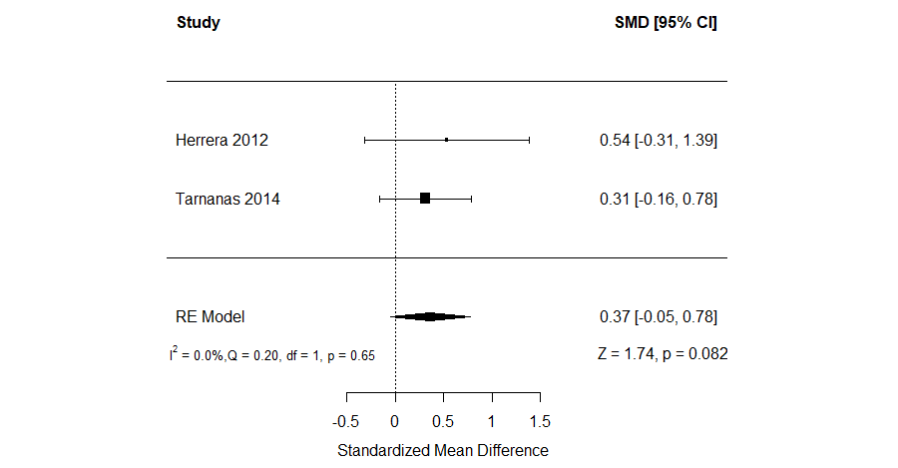
Forest plot of 2 studies comparing the effect of serious games with that of conventional cognitive activities on working memory. RE: random effect; SMD: standardized mean difference [59,61].

The effect of serious games on working memory was compared with that of conventional cognitive activities in 11% (2/18) of the studies [57,59] (Figure 11 [57,59]). A meta-analysis of the results of these studies showed no statistically significant difference (*P*=.08) in working memory between the groups (SMD=0.37, 95% CI −0.05 to 0.78). The statistical heterogeneity of the evidence was not a concern (*P*=.65; *I*^2^=0%). The quality of the evidence was very low as it was downgraded by 3 levels owing to a high risk of bias and imprecision (Multimedia Appendix 10).

##### Serious Games Versus Other Serious Games

The effect of serious games on working memory was compared with that of other serious games in 22% (4/18) of the studies [53,58,61,63]. Specifically, the first study compared the effect of a cognitive training game with that of exergames [53]. The study found a statistically significant difference (*P*<.001) in memory between the groups, favoring cognitive training games over exergames [53]. The second study assessed the effect of 2 cognitive training games on working memory: a recollection training game and a recognition practice game [61]. The study showed no statistically significant difference in working memory between the 2 groups as measured by the n-back task (*P*=.78) and reading span task (*P*=.76) [61].

**Figure 12:**
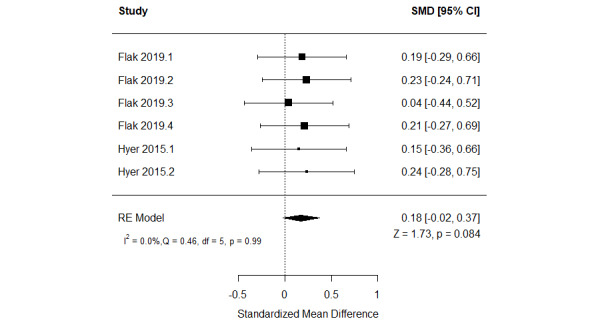
Forest plot of 2 studies (6 comparisons) comparing the effect of adaptive serious games with that of nonadaptive serious games on working memory. RE: random effect; SMD: standardized mean difference [60,65].

The remaining 50% (2/4) of the studies compared the effect of adaptive serious games with that of nonadaptive serious games [58,63]. Of the 2 studies, 1 (50%) assessed working memory using 4 different measures [58], whereas the other study (50%) used 2 different measures to do so [63]. Hence, we included the results of all these measures in the meta-analysis to form 6 comparisons. As shown in Figure 12 [58,63], there was no statistically significant difference (*P*=.08) in working memory between adaptive serious games and nonadaptive serious games (SMD=0.18, 95% CI −0.02 to 0.37). The statistical heterogeneity of the evidence was not a concern (*P*=.99; *I*^2^=0%). The quality of the evidence was low as it was downgraded by 2 levels owing to a high risk of bias and imprecision (Multimedia Appendix 10).

## Discussion

### Principal Findings

This study summarized the evidence regarding the effectiveness of serious games in improving memory. Our meta-analyses showed that serious games are more effective than no or passive interventions in improving nonverbal and working memory. Surprisingly, we found that serious games are as effective as no or passive interventions in improving verbal memory, which, therefore, deems serious games ineffective. This review demonstrated that serious games are more effective than conventional exercises in improving verbal memory. However, we found that serious games are as effective as conventional exercises in improving nonverbal and working memory, indicating that serious games are comparable with conventional exercises. Evidence suggests that cognitive training and exercise work through distinct neuronal mechanisms and, therefore, if combined, they might have synergistic and more effective results compared with being used as separate interventions [65,66]. Studying this synergistic relationship will become important in future primary research and trials. With the advances in virtual reality technologies, their availability, and rising applications of the metaverse [67], more evidence is needed to assess the effectiveness of virtual reality–based exergames in improving memory [68].

The meta-analyses in this review showed that serious games are as effective as conventional cognitive training in improving verbal, nonverbal, and working memory, meaning that serious games and conventional cognitive training are comparable. Furthermore, we found that the effect of adaptive serious games is similar to that of nonadaptive serious games in improving working memory.

The findings of our review and previous reviews were consistent for some outcomes and different for others. Specifically, a systematic review conducted by Lampit et al [32] compared the effect of cognitive training games with that of passive and active interventions on verbal, nonverbal, and working memory in healthy older adults. Consistent with our findings, the review found no statistically significant difference (*P*>.05) in the effect of cognitive training games and of no or passive interventions on verbal memory, and there was a statistically significant difference in working memory between the groups, favoring cognitive training games over no or passive interventions [32]. In contrast to our findings, Lampit et al [32] did not find a statistically significant difference in nonverbal memory between the groups. The contrary finding may be attributed to the following reasons: (1) although the number of participants was ≥100 in 17% (3/18) of the studies in our meta-analysis, the number of participants was <100 in all studies included in the meta-analysis by Lampit et al [32]; (2) the total number of training hours was >15 in only 2 studies included in the meta-analysis by Lampit et al [32], whereas the total number of training hours was >15 in 28% (5/18) of the studies in our meta-analysis; and (3) all studies meta-analyzed in our review (15/18, 83%) recruited participants with cognitive impairment, whereas all studies meta-analyzed in the review by Lampit et al [32] recruited participants without cognitive impairment.

In contrast to our findings, Lampit et al [32] found a statistically significant difference in verbal, nonverbal, and working memory between the groups, favoring cognitive training games over active interventions [32]. This may be attributed to the following reasons: (1) our findings related to these comparisons are based on meta-analyses of 11% (2/18) of the studies, whereas the findings of Lampit et al [32] are based on meta-analyses of 6 to 15 studies; (2) Lampit et al [32] compared cognitive training games with all active interventions, but this review compared cognitive training games with specific active interventions (ie, conventional exercises and conventional cognitive training); (3) all studies meta-analyzed in our review (15/18, 83%) recruited participants with cognitive impairment, whereas all studies meta-analyzed in the review by Lampit et al [32] recruited participants without cognitive impairment; and (4) the review by Lampit et al [32] included some pilot RCTs, whereas our review included only RCTs.

Another review examined the effect of cognitive training games on verbal and working memory among healthy older adults regardless of the comparator type (ie, passive and active controls) [30]. Meta-analyses in that review showed a statistically significant difference in verbal memory (*P*=.03) and working memory (*P*<.001) between the groups, favoring cognitive training games over all types of comparators [30].

Hill et al [34] conducted a systematic review to assess the effect of cognitive training games on verbal, nonverbal, and working memory among people with MCI or dementia regardless of the comparator type. For people with MCI, the review found a statistically significant difference between the groups in verbal and working memory (*P*<.001), favoring all types of comparators [34]. In contrast, there was no statistically significant effect of cognitive training games on nonverbal memory when compared with all types of comparators [34]. For people with dementia, the review showed no statistically significant effect of cognitive training games on verbal, nonverbal, or working memory when compared with all types of comparators [34].

### Research and Practical Implications

#### Research Implications

Given that the review focused on memory among older adults with cognitive impairment, future reviews should assess the effectiveness of serious games on other cognitive functions (eg, learning, language, executive function, and processing speed) in young and older adults with or without cognitive impairment. In this review, a few studies (≤3) were included in the meta-analyses that compared serious games with active interventions (ie, conventional exercises, conventional cognitive training, and nonadaptive serious games); therefore, our findings regarding these comparisons remain inconclusive. Thus, there is a pressing need to conduct more studies to compare the effect of serious games on memory with active interventions.

Most studies in this review (12/18, 67%) were carried out in clinical settings, thus offering the researchers more control over the experiments. However, the participants may have been stressed by playing these games outside the environment that they were used to. Therefore, more studies should be conducted in the community and home settings, allowing participants to be at ease and enabling the researchers to examine other factors that could come into play, such as environmental conditions (eg, room temperature and lighting).

In this review, the long-term effect of serious games was not assessed as few studies reported follow-up data, and the follow-up period was not consistent among the studies. Further studies should assess the long-term effect of serious games on memory. Most of the included studies (15/18, 83%) did not report the mean and SD of pre-post intervention change in memory for each group. Researchers should report this information to accurately calculate effect sizes.

Future studies should also examine and compare the effectiveness of playing serious games in multiplayer mode with other members of the family or community as this has not been assessed in previous studies. We urge researchers to conduct and report RCTs following recommended guidelines or tools (eg, RoB 2 [39]) to avoid the biases identified in this review.

#### Practical Implications

This review shows that serious games can be effective in improving verbal, nonverbal, and working memory. However, these findings should be interpreted cautiously given that most meta-analyses were based on a few studies (≤3) and judged to have a low quality of evidence for the following reasons: most of the included studies (11/18, 61%) were judged to have some concerns regarding the overall bias, the heterogeneity of the evidence was high in approximately half of the meta-analyses (4/10, 40%), and the total effect sizes were imprecise in all meta-analyses (10/10, 100%). On the basis of our review findings, serious games are still not ready as substitutes for real-world interactions and experiences; they should still be used as a supplement rather than an alternative method for interventions targeting the improvement of verbal, nonverbal, and working memory until more evidence suggests otherwise.

Despite the ubiquity and availability of smart mobile devices (ie, tablets and smartphones), only 6% (1/18) of the included studies used them [60]. Mobile devices can be more pervasive and accessible than PCs or commercially available gaming consoles. Studies estimate that, in 2021 alone, approximately 15 billion mobile devices exist worldwide and are used by >7.1 billion users [69]; this is expected to rise. Game and app developers should invest in creating serious games on mobile devices that target improving verbal, nonverbal, and working memory.

### Limitations

This review cannot comment on the effectiveness of serious games (1) delivered on nondigital platforms, (2) used for other purposes (eg, screening or diagnosis), (3) used for improving other cognitive abilities (eg, learning, processing speed, and executive functions), (4) among other age groups, or (5) among those without cognitive impairment. This is because such interventions, outcomes, and populations were beyond the scope of this review.

It is likely that we missed some relevant studies as this review did not search some databases (eg, PubMed and the Cochrane Library [CENTRAL]) and excluded studies that were quasi-experiments, pilot RCTs, published before 2010, or written in non-English languages. The quality of the evidence was very low in all meta-analyses except for 10% (1/10); this may decrease the internal validity of our findings. We cannot comment on the long-term effect of serious games on memory as this review focused on the short-term effect of serious games by meta-analyzing only postintervention data rather than follow-up data. This is because the follow-up period was not consistent among the studies.

The effect size for each meta-analyzed study was likely overestimated or underestimated in this review given that the authors used postintervention data for each group to assess the effect size rather than the pre-post intervention change for each group. Postintervention outcome data were used as most studies (15/18, 83%) did not report the mean and SD for pre-post intervention change in memory for each group, and there was no statistically significant difference in memory between the groups at baseline in all studies (18/18, 100%).

### Conclusions

Serious games may have a significant role to play in improving verbal, nonverbal, and working memory in older adults with cognitive impairment. However, these findings should be treated with caution given that most meta-analyses (7/10, 70%) were based on a few studies (≤3) and judged to have a low quality of evidence for the following reasons: most of the included studies (11/18, 61%) were judged to have some concerns regarding the overall bias, the heterogeneity of the evidence was high in approximately half of the meta-analyses (4/10, 40%), and the total effect sizes were imprecise in all meta-analyses (10/10, 100%). Therefore, serious games should be offered as a supplement to existing proven and safe interventions rather than as a complete substitute until further, more robust evidence is available. Further reviews are necessary to investigate the short- and long-term effect of serious games on memory and other cognitive abilities (eg, executive function, processing speed, and learning) among people of different age groups with or without cognitive impairment.

## Appendix

Multimedia Appendix 1 PRISMA (Preferred Reporting Items for Systematic Reviews and Meta-Analyses) checklist.

Multimedia Appendix 2 Search strategy.

Multimedia Appendix 3 Data extraction form.

Multimedia Appendix 4 Excluded studies.

Multimedia Appendix 5 Reviewers’ judgments about each risk of bias domain for each included study.

Multimedia Appendix 6 Grading of Recommendations Assessment, Development, and Evaluation profile for comparison of serious games with control, conventional exercises, and conventional cognitive activities for verbal memory.

Multimedia Appendix 7 Moderation analyses for verbal memory.

Multimedia Appendix 8 Grading of Recommendations Assessment, Development, and Evaluation profile for comparison of serious games with control, conventional exercises, conventional cognitive activities, and other serious games for nonverbal memory.

Multimedia Appendix 9 Moderation analyses for nonverbal memory.

Multimedia Appendix 10 Grading of Recommendations Assessment, Development, and Evaluation profile for comparison of serious games with control, conventional exercises, conventional cognitive activities, and other serious games for working memory.

Multimedia Appendix 11 Moderation analyses for working memory.

## Abbreviations

BSRT-Delay: Buschke Selective Reminding Test-Delay
MCI: mild cognitive impairment
MCID: minimal clinically important difference
PRISMA: Preferred Reporting Items for Systematic Reviews and Meta-Analyses
RCT: randomized controlled trial
RoB 2: Risk of Bias 2
SMD: standardized mean difference
WMS-III-LM-II: Wechsler Memory Scale Third Edition-Logical Memory II
