# Supplementary material for: The Effectiveness of Serious Games in Improving Memory Among Older Adults With Cognitive Impairment: Systematic Review and Meta-analysis
Source: JMIR Serious Games. 2022 Aug 9;10(3):e35202. doi: 10.2196/35202 (PMC9399845; doi:10.2196/35202)
Supplement: Multimedia Appendix 2 [file games_v10i3e35202_app2.docx]

**Appendix 2 Search strategy**

Database(s): **Ovid MEDLINE(R) ALL**1946 to August 06, 2021
Search Strategy:

| **#** | **Searches** | **Results** |
| --- | --- | --- |
| 1 | exp Cognitive Dysfunction/ | 24059 |
| 2 | "cognitive impair*".tw. | 71868 |
| 3 | "cognitive disorder*".tw. | 4717 |
| 4 | "Cognitive Dysfunction".tw. | 15018 |
| 5 | exp Dementia/ | 178263 |
| 6 | dementia*.tw. | 118309 |
| 7 | exp Alzheimer Disease/ | 101402 |
| 8 | Alzheimer*.tw. | 157327 |
| 9 | exp Randomized Controlled Trials as Topic/ | 150503 |
| 10 | "randomized controlled trial*".tw. | 155117 |
| 11 | "randomised controlled trial*".tw. | 48100 |
| 12 | "randomized control trial*".tw. | 8086 |
| 13 | "randomised control trial*".tw. | 2049 |
| 14 | "clinical trial*".tw. | 406843 |
| 15 | experiment*.tw. | 2176456 |
| 16 | Video Games/ | 6173 |
| 17 | "serious gam*".tw. | 840 |
| 18 | "game-based".tw. | 755 |
| 19 | "videogame*".tw. | 799 |
| 20 | "video game*".tw. | 3657 |
| 21 | "virtual reality game*".tw. | 122 |
| 22 | "virtual reality-based game*".tw. | 3 |
| 23 | "Augmented Reality-based game*".tw. | 0 |
| 24 | "Augmented Reality game*".tw. | 34 |
| 25 | "gamification".tw. | 673 |
| 26 | exergame*.tw. | 559 |
| 27 | "Applied game*".tw. | 22 |
| 28 | 1 or 2 or 3 or 4 or 5 or 6 or 7 or 8 | 328815 |
| 29 | 9 or 10 or 11 or 12 or 13 or 14 or 15 | 2801578 |
| 30 | 16 or 17 or 18 or 19 or 20 or 21 or 22 or 23 or 24 or 25 or 26 or 27 | 9958 |
| 31 | 28 and 29 and 30 | 76 |
| 32 | limit 31 to (english language and yr="2010 -Current") | 75 |

Database(s): **Embase**1996 to 2021 Week 31
Search Strategy:

| **#** | **Searches** | **Results** |
| --- | --- | --- |
| 1 | exp Cognitive Dysfunction/ | 485165 |
| 2 | "cognitive impair*".tw. | 109371 |
| 3 | "cognitive disorder*".tw. | 7361 |
| 4 | "Cognitive Dysfunction".tw. | 22034 |
| 5 | exp Dementia/ | 349095 |
| 6 | dementia*.tw. | 156224 |
| 7 | exp Alzheimer Disease/ | 196990 |
| 8 | Alzheimer*.tw. | 199449 |
| 9 | exp Randomized Controlled Trials as Topic/ | 208422 |
| 10 | "randomized controlled trial*".tw. | 198900 |
| 11 | "randomised controlled trial*".tw. | 62835 |
| 12 | "randomized control trial*".tw. | 12526 |
| 13 | "randomised control trial*".tw. | 3401 |
| 14 | "clinical trial*".tw. | 545714 |
| 15 | experiment*.tw. | 1945882 |
| 16 | Video Games/ | 4364 |
| 17 | "serious gam*".tw. | 952 |
| 18 | "game-based".tw. | 832 |
| 19 | "videogame*".tw. | 955 |
| 20 | "video game*".tw. | 4418 |
| 21 | "virtual reality game*".tw. | 164 |
| 22 | "virtual reality-based game*".tw. | 4 |
| 23 | "Augmented Reality-based game*".tw. | 0 |
| 24 | "Augmented Reality game*".tw. | 29 |
| 25 | "gamification".tw. | 741 |
| 26 | exergame*.tw. | 560 |
| 27 | "Applied game*".tw. | 21 |
| 28 | 1 or 2 or 3 or 4 or 5 or 6 or 7 or 8 | 545244 |
| 29 | 9 or 10 or 11 or 12 or 13 or 14 or 15 | 2788651 |
| 30 | 16 or 17 or 18 or 19 or 20 or 21 or 22 or 23 or 24 or 25 or 26 or 27 | 9669 |
| 31 | 28 and 29 and 30 | 135 |
| 32 | limit 31 to (english language and yr="2010 -Current") | 131 |
| 33 | limit 32 to exclude medline journals | 29 |

Database(s): **APA PsycInfo**2002 to August Week 1 2021
Search Strategy:

| **#** | **Searches** | **Results** |
| --- | --- | --- |
| 1 | exp Cognitive Dysfunction/ | 38290 |
| 2 | "cognitive impair*".tw. | 37180 |
| 3 | "cognitive disorder*".tw. | 2542 |
| 4 | "Cognitive Dysfunction".tw. | 5975 |
| 5 | exp Dementia/ | 63746 |
| 6 | dementia*.tw. | 53233 |
| 7 | exp Alzheimer Disease/ | 38640 |
| 8 | Alzheimer*.tw. | 52230 |
| 9 | exp Randomized Controlled Trials as Topic/ | 0 |
| 10 | "randomized controlled trial*".tw. | 29262 |
| 11 | "randomised controlled trial*".tw. | 5888 |
| 12 | "randomized control trial*".tw. | 2106 |
| 13 | "randomised control trial*".tw. | 397 |
| 14 | "clinical trial*".tw. | 33936 |
| 15 | experiment*.tw. | 265776 |
| 16 | Video Games/ | 7587 |
| 17 | "serious gam*".tw. | 947 |
| 18 | "game-based".tw. | 1548 |
| 19 | "videogame*".tw. | 995 |
| 20 | "video game*".tw. | 5377 |
| 21 | "virtual reality game*".tw. | 67 |
| 22 | "virtual reality-based game*".tw. | 1 |
| 23 | "Augmented Reality-based game*".tw. | 0 |
| 24 | "Augmented Reality game*".tw. | 51 |
| 25 | "gamification".tw. | 764 |
| 26 | exergame*.tw. | 315 |
| 27 | "Applied game*".tw. | 19 |
| 28 | 1 or 2 or 3 or 4 or 5 or 6 or 7 or 8 | 118832 |
| 29 | 9 or 10 or 11 or 12 or 13 or 14 or 15 | 327587 |
| 30 | 16 or 17 or 18 or 19 or 20 or 21 or 22 or 23 or 24 or 25 or 26 or 27 | 11617 |
| 31 | 28 and 29 and 30 | 36 |
| 32 | limit 31 to (english language and yr="2010 -Current") | 33 |

Database(s): **CINHAL (EBSCO)**

| **#** | **Query** | **Results** |
| --- | --- | --- |
| S1 | SU cognitive impairment OR TI "cognitive impair*" OR AB "cognitive impair*" | 25797 |
| S2 | SU cognitive dysfunction [mesh] OR TI "Cognitive Dysfunction" OR AB "Cognitive Dysfunction" | 3581 |
| S3 | SU cognitive disorder* OR TI "cognitive disorder*" OR AB "cognitive disorder* | 4700 |
| S4 | SU Dementia OR TI Dementia OR AB Dementia | 68387 |
| S5 | SU alzheimer's disease OR TI alzheimer's disease OR AB alzheimer's disease | 43547 |
| S6 | SU Randomized Controlled Trials OR TI "Randomized Controlled Trial*" OR AB "Randomized Controlled Trial" | 148077 |
| S7 | TI "Randomised Controlled Trial*" OR AB "Randomised Controlled Trial*" | 25419 |
| S8 | TI "Randomized Control Trial*" OR AB "Randomized Control Trial*" | 3878 |
| S9 | TI "Randomised Control Trial*" OR AB "Randomised Control Trial*" | 1127 |
| S10 | TI "clinical trial*" OR AB "clinical trial*" | 119052 |
| S11 | TI experiment* OR AB experiment* | 146255 |
| S12 | SU Video Games OR TI "Video Game*" OR "Video Game*" | 5809 |
| S13 | SU serious games OR TI "serious gam*" OR AB "serious gam*" | 418 |
| S14 | TI "game-based" OR AB "game-based" | 451 |
| S15 | TI "videogame*" OR AB "videogame*" | 316 |
| S16 | TI "virtual reality game*" OR AB "virtual reality game*" | 68 |
| S17 | TI "Augmented Reality-based game*" OR AB "Augmented Reality-based game*" | 0 |
| S18 | TI "Augmented Reality game*" OR AB "Augmented Reality game*" | 22 |
| S19 | TI "gamification" OR AB "gamification" | 400 |
| S20 | TI exergam* OR AB exergam* | 390 |
| S21 | TI "Applied game*" OR AB "Applied game*" | 10 |
| S22 | S1 OR S2 OR S3 OR S4 OR S5 | 115450 |
| S23 | (S6 OR S7 OR S8 OR S9 OR S10 OR S11) | 393294 |
| S24 | S12 OR S13 OR S14 OR S15 OR S16 OR S17 OR S18 OR S19 OR S20 OR S21 | 6927 |
| S25 | (S12 OR S13 OR S14 OR S15 OR S16 OR S17 OR S18 OR S19 OR S20 OR S21) AND (S22 AND S23 AND S24) | 28 |
| S26 | Limiters - English Language (S25) | 26 |

Database(s): **CENTRAL**

| **#** | **Searches** | **Results** |
| --- | --- | --- |
| 1 | exp Cognitive Dysfunction/ | 2086 |
| 2 | "cognitive impair*".tw. | 10420 |
| 3 | "cognitive disorder*".tw. | 4215 |
| 4 | "Cognitive Dysfunction".tw. | 4067 |
| 5 | exp Dementia/ | 6379 |
| 6 | dementia*.tw. | 14168 |
| 7 | exp Alzheimer Disease/ | 3623 |
| 8 | Alzheimer*.tw. | 11819 |
| 9 | exp Randomized Controlled Trials as Topic/ | 8491 |
| 10 | "randomized controlled trial*".tw. | 580675 |
| 11 | "randomised controlled trial*".tw. | 580675 |
| 12 | "randomized control trial*".tw. | 580675 |
| 13 | "randomised control trial*".tw. | 580675 |
| 14 | "clinical trial*".tw. | 450074 |
| 15 | experiment*.tw. | 101652 |
| 16 | Video Games/ | 789 |
| 17 | "serious gam*".tw. | 290 |
| 18 | "game-based".tw. | 408 |
| 19 | "videogame*".tw. | 353 |
| 20 | "video game*".tw. | 2027 |
| 21 | "virtual reality game*".tw. | 124 |
| 22 | "virtual reality-based game*".tw. | 2 |
| 23 | "Augmented Reality-based game*".tw. | 2 |
| 24 | "Augmented Reality game*".tw. | 0 |
| 25 | "gamification".tw. | 247 |
| 26 | exergame*.tw. | 339 |
| 27 | "Applied game*".tw. | 5 |
| 28 | 1 or 2 or 3 or 4 or 5 or 6 or 7 or 8 | 31886 |
| 29 | 9 or 10 or 11 or 12 or 13 or 14 or 15 | 814178 |
| 30 | 16 or 17 or 18 or 19 or 20 or 21 or 22 or 23 or 24 or 25 or 26 or 27 | 2254 |
| 31 | 28 and 29 and 30 | 152 |
| 32 | limit 31 to (yr="2010 -2021") | 152 |

| **Database** | **Query** | **Results** |
| --- | --- | --- |
| **Scopus** | ( TITLE-ABS-KEY ( "serious gam*" OR "game-based" OR "videogame*" OR "video game*" OR "virtual reality game*" OR "virtual reality-based game*" OR "Augmented Reality-based game*" OR "Augmented Reality game*" OR "gamification" OR gamified OR exergam* OR "Applied game*" ) AND TITLE-ABS-KEY ( "cognitive impair*" OR "cognitive disorder*" OR "Cognitive Dysfunction" OR dementia* OR alzheimer* ) AND TITLE-ABS-KEY ( "randomized controlled trial*" OR "randomised controlled trial*" OR "randomized control trial*" OR "randomised control trial*" OR "clinical trial*" OR experiment*  ) ) AND ( LIMIT-TO ( PUBYEAR , 2021 ) OR LIMIT-TO ( PUBYEAR , 2020 ) OR LIMIT-TO ( PUBYEAR , 2019 ) OR LIMIT-TO ( PUBYEAR , 2018 ) OR LIMIT-TO ( PUBYEAR , 2017 ) OR LIMIT-TO ( PUBYEAR , 2016 ) OR LIMIT-TO ( PUBYEAR , 2015 ) OR LIMIT-TO ( PUBYEAR , 2014 ) OR LIMIT-TO ( PUBYEAR , 2013 ) OR LIMIT-TO ( PUBYEAR , 2012 ) OR LIMIT-TO ( PUBYEAR , 2011 ) OR LIMIT-TO ( PUBYEAR , 2010 ) ) AND ( LIMIT-TO ( LANGUAGE , "English" ) ) AND ( LIMIT-TO ( DOCTYPE , "ar" ) OR LIMIT-TO ( DOCTYPE , "cp" ) ) | 151 |
| **IEEE Xplore** | ("Abstract":"serious game" OR "Abstract":"serious games" OR "Abstract":"game-based" OR "Abstract":"videogames" OR "Abstract":"video games" OR "Abstract":"videogame" OR "Abstract":"video game" OR "Abstract":"virtual reality game" OR "Abstract":"virtual reality games" OR "Abstract":"Augmented Reality game" OR "Abstract":"Augmented Reality games" OR "Abstract":"gamification" OR "Abstract":gamified OR "Abstract":exergam* OR "Abstract":"Applied game") AND ("Abstract":"cognitive impair*" OR "Abstract": "cognitive disorder*" OR "Abstract": "Cognitive Dysfunction" OR "Abstract": dementia OR "Abstract": alzheimer*) | 47 |
| **ACM Digital library** | *[[Abstract: "cognitive impair*"] OR [Abstract: "cognitive disorder*"] OR [Abstract: "cognitive dysfunction"] OR [Abstract: dementia*] OR [Abstract: alzheimer*]] AND [[Abstract: "serious gam*"] OR [Abstract: "game-based"] OR [Abstract: "videogame*"] OR [Abstract: "video game*"] OR [Abstract: "virtual reality game*"] OR [Abstract: "virtual reality-based game*"] OR [Abstract: "augmented reality-based game*"] OR [Abstract: "augmented reality game*"] OR [Abstract: "gamification"] OR [Abstract: gamified] OR [Abstract: exergam*] OR [Abstract: "applied game*"]] AND [[All: "randomized controlled trial*"] OR [All: "randomised controlled trial*"] OR [All: "randomized control trial*"] OR [All: "randomised control trial*"] OR [All: "clinical trial*"] OR [All: experiment*]] AND [Publication Date: (01/01/2010 TO 12/31/2021)]* | 5 |
| **Google Scholar** | ("cognitive impair*" OR "cognitive disorder*" OR "Cognitive Dysfunction" OR dementia* OR alzheimer*) AND ("serious gam*" OR "game-based" OR exergam*) AND ("controlled trial*" OR "control trial*") | 100 |

"cognitive impair*" OR "cognitive disorder*" OR "Cognitive Dysfunction" OR dementia* OR Alzheimer*

"randomized controlled trial*" OR "randomised controlled trial*" OR "randomized control trial*" OR "randomised control trial*" OR "clinical trial*" OR experiment*

"serious game*" OR "game-based" OR "videogame*" OR "video game*" OR "virtual reality game*" OR "virtual reality-based game*" OR "Augmented Reality game*" OR "Augmented Reality-based game*" OR gamification OR exergame* OR "Applied game*"
