# Supplementary figures and images for: The Effectiveness of Serious Games in Improving Memory Among Older Adults With Cognitive Impairment: Systematic Review and Meta-analysis
Source: JMIR Serious Games. 2022 Aug 9;10(3):e35202. doi: 10.2196/35202 (PMC9399845; doi:10.2196/35202)

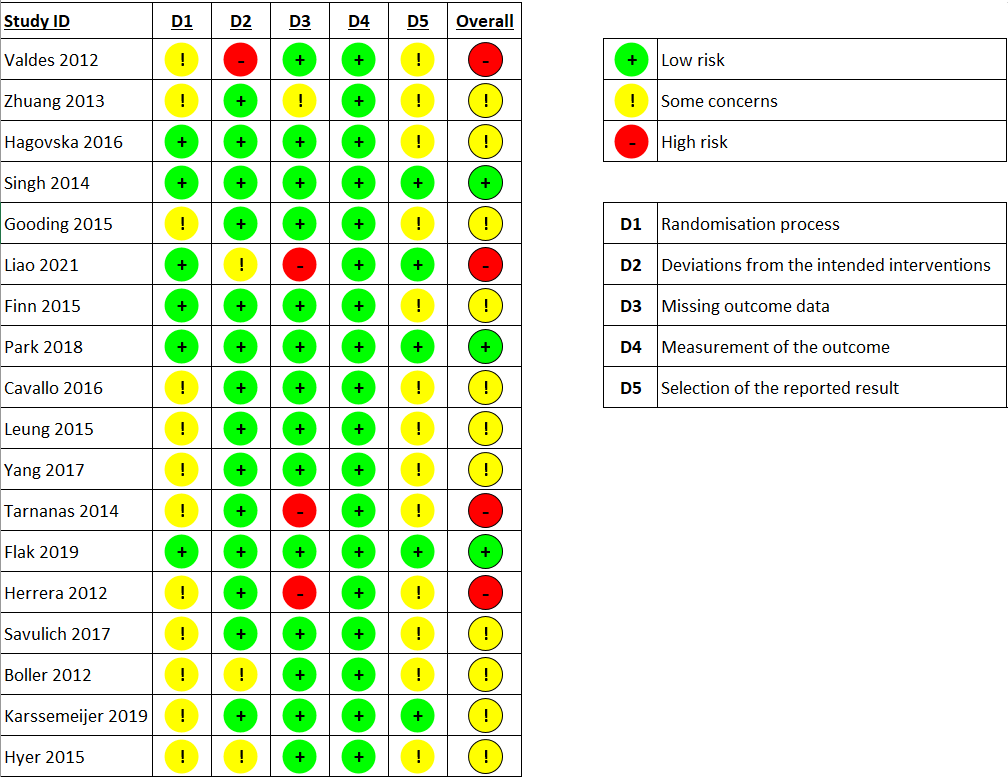
**Appendix 5 Reviewers’ judgements about each “risk of bias” domain for each included study**

Supplement: Multimedia Appendix 5 [file games_v10i3e35202_app5.docx]
