# Supplementary material for: The Effectiveness of Serious Games in Improving Memory Among Older Adults With Cognitive Impairment: Systematic Review and Meta-analysis
Source: JMIR Serious Games. 2022 Aug 9;10(3):e35202. doi: 10.2196/35202 (PMC9399845; doi:10.2196/35202)
Supplement: Multimedia Appendix 7 [file games_v10i3e35202_app7.docx]

**Appendix 7: Moderation analyses for verbal memory**

**Sample size: <100 vs. ≥100**

|  | **Estimate** | **SE** | **Z-value** | **P-value** | **95% CI** |
| --- | --- | --- | --- | --- | --- |
| **mods** | -0.6837 | 0.4481 | -1.5256 | 0.1271 | -1.5620 to 0.1947 |

**Health condition: MCI vs. AD**

|  | **Estimate** | **SE** | **Z-value** | **P-value** | **95% CI** |
| --- | --- | --- | --- | --- | --- |
| **mods** | 1.3064 | 0.4454 | 2.9331 | **0.0034** | 0.4335 to 2.1794 |

**Setting: Clinical vs. Community**

|  | **Estimate** | **SE** | **Z-value** | **P-value** | **95% CI** |
| --- | --- | --- | --- | --- | --- |
| **mods** | -0.6504 | 0.4627 | -1.4058 | 0.1598 | -1.5572 to 0.2564 |

**Type of serious games: Supervised vs. Unsupervised**

|  | **Estimate** | **SE** | **Z-value** | **P-value** | **95% CI** |
| --- | --- | --- | --- | --- | --- |
| **mods** | -0.6674 | 0.4681 | -1.4256 | 0.1540 | -1.5849 to 0.2501 |

**Duration: ≤60 vs. >60**

|  | **Estimate** | **SE** | **Z-value** | **P-value** | **95% CI** |
| --- | --- | --- | --- | --- | --- |
| **mods** | 0.3597 | 0.6022 | 0.5974 | 0.5503 | -0.8205 to 1.5399 |

**Frequency: Two times vs. Three times**

|  | **Estimate** | **SE** | **Z-value** | **P-value** | **95% CI** |
| --- | --- | --- | --- | --- | --- |
| **mods** | 0.3100 | 0.5376 | 0.5765 | 0.5642 | -0.7438 to 1.3637 |

**Period: ≤12 weeks vs. >12 weeks**

|  | **Estimate** | **SE** | **Z-value** | **P-value** | **95% CI** |
| --- | --- | --- | --- | --- | --- |
| **mods** | -0.8880 | 0.4516 | -1.9664 | **0.050** | -1.7730 to -0.0029 |
