# Supplementary material for: The Effectiveness of Serious Games in Improving Memory Among Older Adults With Cognitive Impairment: Systematic Review and Meta-analysis
Source: JMIR Serious Games. 2022 Aug 9;10(3):e35202. doi: 10.2196/35202 (PMC9399845; doi:10.2196/35202)
Supplement: Multimedia Appendix 8 [file games_v10i3e35202_app8.docx]

| **Appendix 8 GRADE Profile for comparison of serious games to control, conventional exercises,** **conventional cognitive activities for non-verbal memory** | | | | | | | | | | | |
| --- | --- | --- | --- | --- | --- | --- | --- | --- | --- | --- | --- |
| **Certainty assessment** | | | | | | | **Summary of findings** | | | | |
| **Participants (studies) Follow-up** | **Risk of bias** | **Inconsistency** | **Indirectness** | **Imprecision** | **Publication bias** | **Overall certainty of evidence** | **Study event rates (%)** | | **Relative effect (95% CI)** | **Anticipated absolute effects** | |
|  |  |  |  |  |  |  | **Control** | **Serious games** |  | **Risk with** | **Risk difference with** |
| **Serious games vs. Control** | | | | | | | | | | | |
| 843 (8 RCTs)  (10 comparisons) | very serious^a^ | very serious^b^ | not serious | serious^c,d^ | none | ⨁◯◯◯ Very low | 416 | 427 | - | - | SMD **0.46 higher** (0.09 higher to 0.83 higher) |
| **Serious games vs. Exercises** | | | | | | | | | | | |
| 123 (2 RCTs) | very serious^e^ | not serious | not serious | serious^c,f^ | none | ⨁◯◯◯ Very low | 61 | 62 | - | - | SMD **0.19 lower** (0.54 lower to 0.17 higher) |
| **Serious games vs. Conventional cognitive activities** | | | | | | | | | | | |
| 164 (2 RCTs)  (3 comparisons) | very serious^g^ | not serious | not serious | very serious^h,i^ | none | ⨁◯◯◯ Very low | 89 | 75 | - | - | SMD **0.01 lower** (0.32 lower to 0.3 higher) |

**CI:** confidence interval; **SMD:** standardised mean difference

#### Explanations

a. Evidence was downgraded by 2 levels because the overall risk of bias was rated as low in only one study due to issues mainly in the randomization process and selection of the reported results in the remaining studies.

b. Evidence was downgraded by 2 levels as P<0.001 and I^2^=80.1%, indicating high heterogeneity.

c. Evidence was downgraded by 1 level because 95% CI crosses one of MID boundaries for this outcome.

d. MID for this outcome, calculated as ± 0.5 times the standardized mean difference (SMD), is ± 0.23.

e. Evidence was downgraded by 2 levels because there were some concerns in 2 studies due to issues mainly in the randomization process.

f. MID for this outcome, calculated as ± 0.5 times the standardized mean difference (SMD), is ± 0.10.

g. Evidence was downgraded by 2 levels because the overall risk of bias was rated as high in all studies due to issues mainly in the randomization process, missing outcome data, and selection of the reported results.

h. Evidence was downgraded by 2 levels because 95% CI crosses the two MID boundaries for this outcome.

i. MID for this outcome, calculated as ± 0.5 times the standardized mean difference (SMD), is ± 0.005.
