# Supplementary material for: The Effectiveness of Serious Games in Improving Memory Among Older Adults With Cognitive Impairment: Systematic Review and Meta-analysis
Source: JMIR Serious Games. 2022 Aug 9;10(3):e35202. doi: 10.2196/35202 (PMC9399845; doi:10.2196/35202)
Supplement: Multimedia Appendix 9 [file games_v10i3e35202_app9.docx]

**Appendix 9: Moderation analyses for non-verbal memory**

**Sample size: <100 vs. ≥100**

|  | **Estimate** | **SE** | **Z-value** | **P-value** | **95% CI** |
| --- | --- | --- | --- | --- | --- |
| **mods** | -0.5293 | 0.3342 | -1.5840 | 0.1132 | -1.1843 to 0.1256 |

**Health condition: MCI vs. AD**

|  | **Estimate** | **SE** | **Z-value** | **P-value** | **95% CI** |
| --- | --- | --- | --- | --- | --- |
| **mods** | 0.2678 | 0.3678 | 0.7280 | 0.4666 | -0.4532 to 0.9888 |

**Setting: Clinical vs. Community**

|  | **Estimate** | **SE** | **Z-value** | **P-value** | **95% CI** |
| --- | --- | --- | --- | --- | --- |
| **mods** | -0.0418 | 0.1823 | -0.2293 | 0.8187 | -0.3992 to 0.3156 |

**Type of serious games: Supervised vs. Unsupervised**

|  | **Estimate** | **SE** | **Z-value** | **P-value** | **95% CI** |
| --- | --- | --- | --- | --- | --- |
| **mods** | -0.5771 | 0.3876 | -1.4888 | 0.1365 | -1.3367 to 0.1826 |

**Duration: ≤60 vs. >60**

|  | **Estimate** | **SE** | **Z-value** | **P-value** | **95% CI** |
| --- | --- | --- | --- | --- | --- |
| **mods** | 0.2173 | 0.4296 | 0.5058 | 0.6130 | -0.6246 to 1.0592 |

**Frequency: Two times vs. Three times**

|  | **Estimate** | **SE** | **Z-value** | **P-value** | **95% CI** |
| --- | --- | --- | --- | --- | --- |
| **mods** | -0.1995 | 0.3710 | -0.5378 | 0.5907 | -0.9267 to 0.5277 |

**Period: ≤12 weeks vs. >12 weeks**

|  | **Estimate** | **SE** | **Z-value** | **P-value** | **95% CI** |
| --- | --- | --- | --- | --- | --- |
| **mods** | -0.0993 | 0.3832 | -0.2591 | 0.7955 | -0.8504 to 0.6518 |
