# Supplementary material for: The Effectiveness of Serious Games in Improving Memory Among Older Adults With Cognitive Impairment: Systematic Review and Meta-analysis
Source: JMIR Serious Games. 2022 Aug 9;10(3):e35202. doi: 10.2196/35202 (PMC9399845; doi:10.2196/35202)
Supplement: Multimedia Appendix 11 [file games_v10i3e35202_app11.docx]

**Appendix 11: Moderation analyses for working memory**

**Sample size: <100 vs. ≥100**

|  | **Estimate** | **SE** | **Z-value** | **P-value** | **95% CI** |
| --- | --- | --- | --- | --- | --- |
| **mods** | -0.2222 | 0.2923 | -0.7602 | 0.4472 | -0.7951 to 0.3507 |

**Health condition: MCI vs. AD**

|  | **Estimate** | **SE** | **Z-value** | **P-value** | **95% CI** |
| --- | --- | --- | --- | --- | --- |
| **mods** | 0.0073 | 0.3368 | 0.0216 | 0.9828 | -0.6529 to 0.6674 |

**Setting: Clinical vs. Community**

|  | **Estimate** | **SE** | **Z-value** | **P-value** | **95% CI** |
| --- | --- | --- | --- | --- | --- |
| **mods** | -0.0909 | 0.3619 | -0.2511 | 0.8017 | -0.8002 to 0.6184 |

**Type of serious games: Supervised vs. Unsupervised**

|  | **Estimate** | **SE** | **Z-value** | **P-value** | **95% CI** |
| --- | --- | --- | --- | --- | --- |
| **mods** | -0.1215 | 0.3419 | -0.3555 | 0.7222 | -0.7916 to 0.5485 |

**Frequency: Two times vs. Three times**

|  | **Estimate** | **SE** | **Z-value** | **P-value** | **95% CI** |
| --- | --- | --- | --- | --- | --- |
| **mods** | -0.1084 | 0.3210 | -0.3376 | 0.7356 | -0.7375 to 0.5207 |

**Period: ≤12 weeks vs. >12 weeks**

|  | **Estimate** | **SE** | **Z-value** | **P-value** | **95% CI** |
| --- | --- | --- | --- | --- | --- |
| **mods** | 0.1439 | 0.3444 | 0.4179 | 0.6760 | -0.5311 to 0.8190 |
